# PLCE1 polymorphisms and expression combined with serum AFP level predicts survival of HBV-related hepatocellular carcinoma patients after hepatectomy

**DOI:** 10.18632/oncotarget.16346

**Published:** 2017-03-18

**Authors:** Xiwen Liao, Chuangye Han, Wei Qin, Xiaoguang Liu, Long Yu, Guangzhi Zhu, Tingdong Yu, Sicong Lu, Hao Su, Zhen Liu, Zhiwei Chen, Chengkun Yang, Ketuan Huang, Zhengtao Liu, Yu Liang, Jianlu Huang, Jiahong Dong, Lequn Li, Xue Qin, Xinping Ye, Kaiyin Xiao, Minhao Peng, Tao Peng

**Affiliations:** ^1^ Department of Hepatobiliary Surgery, The First Affiliated Hospital of Guangxi Medical University, Nanning, 530021, Guangxi Province, China; ^2^ Department of Hepatobiliary Surgery, Affiliated Hospital of Guangdong Medical University, Zhanjiang, 524001, Guangdong Province, China; ^3^ Department of Hepatobiliary and Pancreatic Surgery, The First Affiliated Hospital of Zhengzhou University, Zhengzhou, 450000, Henan Province, China; ^4^ Department of Hepatobiliary Surgery, The Third Affiliated Hospital of Guangxi Medical University, Nanning, 530031, Guangxi Province, China; ^5^ Department of Hepato-Biliary-Pancreatic Surgery, Beijing Tsinghua Changgung Hospital, Beijing, 102218, China; ^6^ Department of Hepatobiliary Surgery, Affiliated Tumor Hospital of Guangxi Medical University, Nanning, 530021, Guangxi Province, China; ^7^ Department of Clinical Laboratory, The First Affiliated Hospital of Guangxi Medical University, Nanning, 530021, Guangxi Province, China

**Keywords:** hepatocellular carcinoma, hepatitis B virus, PLCE1, AFP, prognosis

## Abstract

Polymorphisms in the *phospholipase C epsilon* (*PLCE*) *1* gene play a crucial role in the development and progression of several types of cancer. The present study investigated the prognostic significance of *PLCE1* gene polymorphisms and expression combined with serum α-fetoprotein (AFP) level in hepatitis B virus (HBV)-related hepatocellular carcinoma (HCC). Single nucleotide polymorphisms were genotyped by sequencing DNA isolated from surgically resected tumor samples of 421 HBV-related HCC patients, and expression profiles were generated based on the GSE14520 dataset. A joint-effects analysis of *PLCE1* haplotypes (A_rs2274223_C_rs3765524_; G_rs2274223_T_rs3765524_) with AFP level stratified at 20 ng/ml showed a significant association with overall survival(OS) of HBV-related HCC patients(log-rank *P*=0.0003). Patients with AC and GT haplotypes with AFP level ≥ 20 ng/ml had an increased risk of death as compared to those with the AC haplotype and AFP level < 20 ng/ml (adjusted *P*=0.029 and 0.041, respectively). Patients with the GT haplotype and AFP level < 20 ng/ml also had an increased risk of death, although with a non-significant P value (adjusted *P*=0.092). Joint-effects analysis of *PLCE1* mRNA expression with serum AFP level stratified at 300 ng/ml was significantly associated with HBV-related HCC recurrence and OS. Our results demonstrate that PLCE1 haplotypes (including rs2274223 and rs3765524) and expression combined with serum AFP level may predict postoperative outcome of HBV-related HCC patients.

## INTRODUCTION

Eastern Asia has the highest incidence of liver cancer in the world [1]. Hepatitis B virus (HBV) infection has a high prevalence (> 5%) in the Chinese population [2, 3], and in 2012, more than half of new cases of liver cancer and death from the disease occurred in China [1]. Liver cancer is the third leading cause of cancer-related death in China [4], with an age-standardized 5-year relative survival rate of 10.1% [5]. Most of these are cases of hepatocellular carcinoma(HCC) [6]. In Guangxi province, which has a higher prevalence of HBV infection, aflatoxin B1 (AFB1) exposure levels and *tumor protein p53* (*TP53*) codon 249 mutation rates are higher than in other provinces, and are accompanied by higher mortality and morbidity from HCC [7–11]. Alcohol abuse, AFB1 exposure, HBV and hepatitis C virus infection are the major environmental factors associated with HCC [12, 13], while *TP53* mutation has been linked to HCC development and prognosis [14–17]. Thus, the population in Guangxi is suitable for exploring the relationship between AFB1 exposure, HBV infection, *TP53* codon 249 mutation, and HCC.

Alpha-fetoprotein (AFP) is a HCC biomarker that has been used to screen high-risk populations as well as for diagnosis, prognosis, and predicting recurrence. However, recent studies have shown that serum AFP levels lack diagnostic and/or prognostic specificity and sensitivity for HCC [18, 19]. As such, HCC guidelines of European Association for the Study of the Liver [20] and the American Association for the Study of Liver Diseases [21] no longer recommend serum AFP measurement; however, a recent clinical study in China suggested that it is still a valuable biomarker for HBV-related HCC [22]. It was also shown that HBVx protein can induce AFP expression in liver cells [23–25]. These findings suggest that AFP is closely associated with HBV and still valuable in HBV-related HCC. Given the high prevalence of HBV infection in China, serum AFP remains the most highly recommended biomarker for HCC diagnosis and prognosis according to Chinese HCC guidelines [26].

*Phospholipase C epsilon* (*PLCE*) *1* single nucleotide polymorphisms (SNPs) rs2274223 and rs3765524 have been identified in many cancer risk studies [27] and genome-wide association studies (GWAS) [28]. Rs2274223 A>G has been linked to altered *PLCE1* expression in esophageal squamous cell carcinoma(ESCC) [29–31], and the G allele may contribute to increased cancer incidence [30]. Our previous GWAS of Chinese HBV-related HCC patients in Guangxi revealed that *PLCE1* gene polymorphism was associated with HBV-related HCC [32]. In our current study, we investigated the utility of *PLCE1* gene polymorphisms and expression in combination with serum AFP levels for predicting the prognosis of HBV-related HCC.

## RESULTS

### Patient characteristics and clinical outcomes

Patients were followed up after surgery until death or the final follow-up, which was in September 2014. A total of 421 HBV-related HCC patients completed the follow-up period successfully, with a lost to follow-up rate of 6.4%. The duration of follow up ranged from 12 to 117 months and the median survival time was 51 months. At the time of analysis, 188 (44.7%) of the patients had died. Clinical characteristics of patients and their association with overall survival (OS)are summarized in Table 1. The Kaplan-Meier analysis revealed that tumor size and number, Barcelona Clinic Liver Cancer (BCLC) stage, and portal vein tumor thrombus (PVTT) were significantly associated with OS (log-rank test, *P* < 0.001)and increased risk of death. In 364 patients (86.4%), a Child–Pugh score of A was related to OS (log-rank test, *P*=0.006). In 240 patients (57%), radical resection was related to OS (log-rank test, *P*=0.033). In 150 patients (35.6%), antiviral therapy after hepatectomy was associated with OS (log-rank test, *P*=0.004) as compared to those who did not receive the therapy. Other clinical characteristics were not associated with OS.

**Table 1:** Clinical characteristics of HBV-related HCC patients

Variables	Patients(n=421)	No. of events (%)	MST(months)	HR (95% CI)	Log-rank P
**Age (years)**					0.186
**≤60**	367	162 (44.1)	58	1	
**>60**	54	25 (46.3)	39	1.326 (0.869-2.025)	
**Gender**					0.274
**Male**	371	169 (45.6)	51	1	
**Female**	50	18 (36.0)	51	0.764 (0.470-1.243)	
**Ethnicity**					0.978
**Han**	259	117 (45.2)	51	1	
**Minority**	162	70 (43.2)	51	1.004 (0.746-1.353)	
**BMI**					0.683
**≤25**	332	145 (43.7)	51	1	
**>25**	89	42 (47.2)	51	0.931 (0.659-1.315)	
**Smoking status**					0.124
**None**	272	115 (42.3)	61	1	
**Ever**	149	72 (48.3)	40	1.259 (0.937-1.692)	
**Drinking status**					0.608
**None**	255	109 (42.7)	51	1	
**Ever**	166	78 (47.0)	45	1.079 (0.806-1.443)	
**Child–Pugh score**					0.006
**A**	364	157 (43.1)	58	1	
**B**	57	30 (52.6)	34	1.718 (1.161-2.542)	
**Cirrhosis**					0.225
**No**	47	18 (38.3)	88	1	
**Yes**	374	169 (45.2)	51	1.348 (0.829-2.193)	
**Radical resection&**					0.033
**Yes**	240	96 (40.0)	73	1	
**None**	171	86 (50.3)	40	1.368 (1.023-1.831)	
**Portal hypertension†**					0.595
**No**	217	103 (47.5)	57	1	
**Yes**	181	77 (42.5)	45	1.084 (0.804-1.462)	
**Pathological diagnosis‡**					0.974
**Well differentiated**	23	10 (43.5)	47	1	
**Moderately differentiated**	323	143 (44.3)	51	1.074 (0.565-2.040)	
**Poorly differentiated**	12	5 (41.7)	40	1.034 (0.353-3.027)	
**Adjuvant antiviral treatment**					0.004
**No**	271	146 (53.9)	41	1	
**Yes**	150	41 (27.3)	NA	0.605 (0.426-0.858)	
**Tumor behavior**					
**Tumor size (cm)**					<0.001
**<10**	316	126 (39.9)	71	1	
**≥10**	105	61 (58.1)	34	1.925 (1.414-2.621)	
**Tumor number**					<0.001
**Single**	309	120 (38.8)	61	1	
**Multiple**	112	67 (59.8)	28	1.891 (1.401-2.551)	
**Regional invasion**					0.068
**Absence**	358	158 (44.1)	58	1	
**Presence**	63	29 (46.0)	40	1.445 (0.968-2.156)	
**BCLC stage**					<0.001
**A**	250	81 (32.4)	95	1	
**B**	69	40 (58.0)	39	2.115 (1.447-3.091)	
**C**	102	66 (64.7)	25	3.216 (2.312-4.473)	
**PVTT**					<0.001
**No**	352	138 (39.2)	71	1	
**Yes**	69	49 (71.0)	19	2.970 (2.135-4.131)	

Notes: & Information of radical resection was unavailable in 10 patients; † Information of portal hypertension was unavailable in 23 patients; ‡ Information of pathological diagnosis was unavailable in 63 patients; BMI, body mass index; BCLC, Barcelona Clinic Liver Cancer; PVTT, portal vein tumor thrombus; MST, median survival time; HR, hazard ratio; CI, confidence interval.

### Bioinformatics analysis

**Figure 1:**
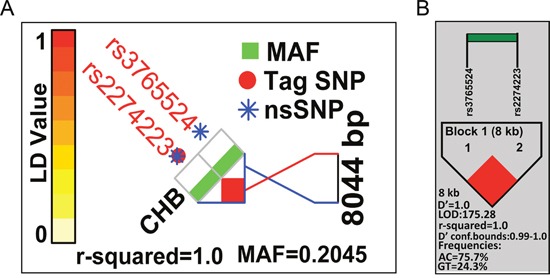
Patterns of LD plots for two selected SNPs in the *PLCE1* gene **(A)** Pattern of LD plot and pairwise LD (r^2^) value calculated based on HapMap data of CHB samples. **(B)** Pattern of LD plot and pairwise LD (r^2^) value calculated based on data from the current study of HBV-related HCC cases.

The success rate for genotyping both SNPs was 100% (Supplementary Figure 1 and 2). The genotype frequencies of rs2274223 and rs3765524 were consistent with Hardy-Weinberg equilibrium(χ^2^=3.091, *P*=0.079). A haplotype analysis of the two selected SNPs in HBV-related HCC patients and the normal Chinese Han in Beijing (CHB) population revealed a haplotype block (block pairwise r^2^=1.0 for normal CHB population; block pairwise r^2^=1.0, A_rs2274223_C_rs3765524_=75.7% and G_rs2274223_T_rs3765524_=24.3% for HBV-related HCC patients) (Figure 1A, 1B). Both SNPs were non-synonymous (rs2274223: H1927R; rs3765524: T1777I) and located in exons; rs2274223 was a Tag SNP of *PLCE1*. In addition, both SNPs were predicted to be ‘Benign’ and ‘Tolerated’ by PolyPzhen-2 (http://genetics.bwh.harvard.edu/pph2/index.shtml) and SIFT (http://sift.jcvi.org/) computational tools.

### Analysis of genetic polymorphisms and haplotypes for different serum AFP levels

The genotype distributions of rs2274223 and rs3765524 for different serum AFP levels are shown in Table 2. In the codominant genetic model, after adjusting for the Child–Pugh score, radical resection, antiviral therapy after hepatectomy, tumor size, tumor number, BCLC stage, PVTT, and regional invasion, single-locus analyses revealed that genotype GG of *PLCE1* rs2274223 was associated with an increased risk for AFP cut-off levels of 200 and 400 ng/ml in HBV-related HCC (GG vs. AA; adjusted *P*=0.013, adjusted odds ratio [OR]=3.014, 95% confidence interval [CI]=1.258–7.224 for an AFP cut-off level of 200 ng/ml; and adjusted *P*=0.020, adjusted OR=2.686, 95% CI=1.169–6.173 for an AFP cut-off level of 400 ng/ml) as compared to genotype AA. In the dominant genetic model of rs2274223, G (AG/GG) allele carriers also showed increased risk in HBV-related HCC with AFP cut-off levels of 200 and 400 ng/ml (AG/GG vs. AA; adjusted *P*=0.024, adjusted OR= 1.607, 95% CI= 1.065–2.424 for an AFP cut-off level of 200 ng/ml; and adjusted *P*= 0.028, adjusted OR= 1.588, 95% CI= 1.052–2.399 for an AFP cut-off level of 400 ng/ml) as compared to AA carriers. The results for rs3765524 were the same as for rs2274223; that is, the T (CT/TT) allele was associated with an increased risk for AFP cut-off levels of 200 and 400 ng/ml as compared to CC. In the haplotype analysis, the GT haplotype was associated with an increased risk for AFP cut-off levels of 200 and 400 ng/ml in HBV-related HCC (GT vs. AC; adjusted P=0.004, adjusted OR= 1.629, 95% CI= 1.164–2.281 for an AFP cut-off level of 200 ng/ml; and adjusted P= 0.006, adjusted OR= 1.592, 95% CI= 1.140–2.224 for an AFP cut-off level of 400 ng/ml). Both rs2274223 and rs3765524 genotype distributions and haplotype analysis results were non-significant for an AFP cut-off level of 20 ng/ml.

**Table 2:** Genotype and haplotype distributions of PLCE1 at different serum AFP levels in HBV-related HCC patients

Variables	AFP (ng/ml)		Crude OR (95%CI)	Crude P	Adjusted OR (95%CI)	Adjusted P§	AFP (ng/ml)		Crude OR (95%CI)	Crude P	Adjusted OR (95%CI)	Adjusted P§	AFP (ng/ml)		Crude OR (95%CI)	Crude P	Adjusted OR (95%CI)	Adjusted P§
	<20	≥20					<200	≥200					<400	≥400				
**Genotypes**																		
**rs2274223**																		
**AA**	78	177	1		1		138	117	1		1		152	103	1		1	
**AG**	35	102	1.284(0.805-2.049)	0.294	1.357(0.833-2.210)	0.22	63	74	1.385(0.913-2.101)	0.125	1.424(0.922-2.198)	0.111	70	67	1.412(0.930-2.146)	0.105	1.427(0.922-2.209)	0.111
**GG**	6	23	1.689(0.662-4.312)	0.273	1.672(0.634-4.408)	0.298	8	21	3.096(1.322-7.249)	0.009	3.014(1.258-7.224)	0.013	10	19	2.804(1.253-6.275)	0.012	2.686(1.169-6.173)	0.02
**AG+GG**	41	125	1.344(0.864-2.090)	0.190	1.403(0.884-2.228)	0.15	71	95	1.578(1.064-2.340)	0.023	1.607(1.065-2.424)	0.024	80	86	1.586(1.070-2.353)	0.022	1.588(1.052-2.399)	0.028
**rs3765524**																		
**CC**	78	177	1		1		138	117	1		1		152	103	1		1	
**CT**	35	102	1.284(0.805-2.049)	0.294	1.357(0.833-2.210)	0.22	63	74	1.385(0.913-2.101)	0.125	1.424(0.922-2.198)	0.111	70	67	1.412(0.930-2.146)	0.105	1.427(0.922-2.209)	0.111
**TT**	6	23	1.689(0.662-4.312)	0.273	1.672(0.634-4.408)	0.298	8	21	3.096(1.322-7.249)	0.009	3.014(1.258-7.224)	0.013	10	19	2.804(1.253-6.275)	0.012	2.686(1.169-6.173)	0.02
**CT+TT**	41	125	1.344(0.864-2.090)	0.190	1.403(0.884-2.228)	0.15	71	95	1.578(1.064-2.340)	0.023	1.607(1.065-2.424)	0.024	80	86	1.586(1.070-2.353)	0.022	1.588(1.052-2.399)	0.028
**Haplotypes**																		
**AC**	191	456	1		1		339	308	1		1		374	273	1		1	
**GT**	47	148	1.319(0.912-1.908)	0.142	1.352(0.922-1.983)	0.123	79	116	1.616(1.168-2.237)	0.004	1.629(1.164-2.281)	0.004	90	105	1.598(1.158-2.205)	0.004	1.592(1.140-2.224)	0.006

Notes: § Adjustment for Child–Pugh score, tumor size, tumor number, BCLC stage, radical resection, regional invasion, adjuvant antiviral treatment, PVTT in logistic regression model; OR, odds ratio; CI, confidence interval.

### Association between haplotypes and clinical features

The association between *PLCE1* haplotypes and clinicopathological characteristics are shown in Table 3. With the exception of Child–Pugh score, none of the associations between risk factors and *PLCE1* haplotypes reached statistical significance. A Child–Pugh score of B was significantly associated with haplotypes AC and GT, suggesting that the haplotype distribution of *PLCE1* differed according to liver functional reserve status in HBV-related HCC patients.

**Table 3:** Association between risk factors and PLCE1 haplotypes in HBV-related HCC patients

Variables	AC(2n=842)	GT(2n=842)	OR (95%CI)	P
**Tumor size(cm)**				
**≤10**	494	138	1	
**≥10**	153	57	1.334 (0.932-1.908)	0.115
**Tumor number**				
**Single**	471	147	1	
**Multiple**	176	48	0.874 (0.604-1.264)	0.474
**Child–Pugh score**				
**A**	548	180	1	
**B**	99	15	0.461 (0.261-0.814)	0.008
**BCLC stage**				
**A**	380	120	1	
**B**	112	26	0.735 (0.458-1.180)	0.203
**C**	155	49	1.001 (0.684-1.466)	0.996
**Radical resection&**				
**Yes**	365	115	1	
**None**	264	78	0.938 (0.675-1.302)	0.701
**Regional invasion**				
**Absence**	557	159	1	
**Presence**	90	36	1.401 (0.916-2.143)	0.12
**PVTT**				
**No**	542	162	1	
**Yes**	105	33	1.051(0.685-1.614)	0.818
**Pathological diagnosis‡**				
**Well differentiated**	31	15	1	
**Moderately differentiated**	502	144	0.593 (0.311-1.128)	0.111
**Poorly differentiated**	19	5	0.544 (0.170-1.739)	0.304

Notes: & Information of radical resection was unavailable in 10 patients; ‡ Information of pathological diagnosis was unavailable in 63 patients; OR, odds ratio; CI, confidence interval; BCLC, Barcelona Clinic Liver Cancer stage; PVTT, portal vein tumor thrombus.

### Relationship between haplotype and clinical outcome

**Figure 2:**
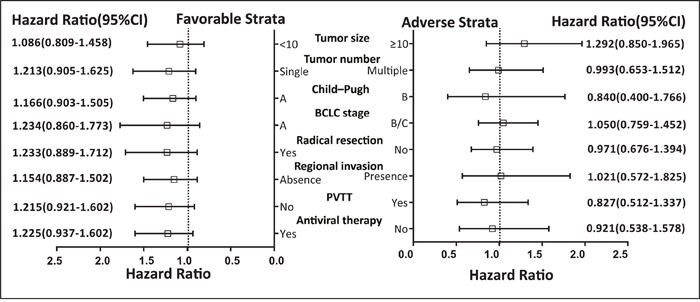
Stratified analysis of association between *PLCE1* haplotype and OS in HBV-related HCC patients Variables were stratified according to favorable and adverse strata.

Results of the stratified analysis between *PLCE1* haplotypes and OS in HBV-related HCC patients are shown in Figure 2. All variables were stratified according to favorable and adverse clinicopathological characteristics. We found that *PLCE1* haplotypes were not associated with OS according to this stratification.

**Figure 3:**
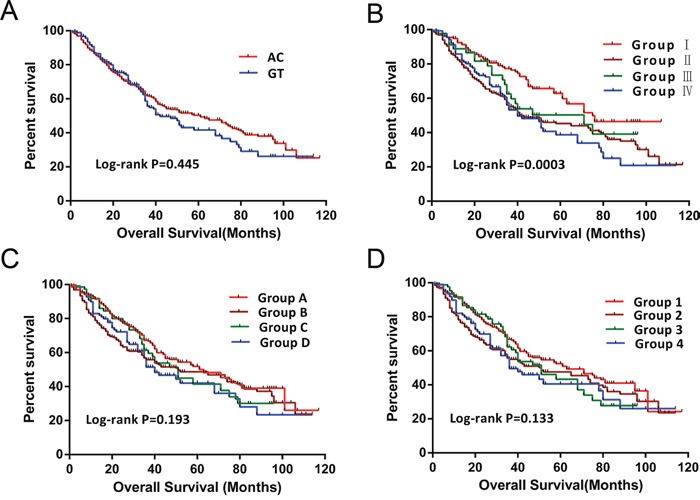
Survival curves of patients with different *PLCE1* haplotypes and joint-effects analysis of different AFP levels **(A)** OS stratified by AC and GT haplotypes. **(B–D)** OS stratified by joint-effects analysis of PLCE1 haplotypes and an AFP cut-off level of 20 ng/ml **(B)**, 200 ng/ml **(C)**, and 400 ng/ml **(D)**.

The univariate analysis of *PLCE1* haplotypes revealed that patients with the GT haplotype had a shorter median survival time (MST) relative to those with the AC haplotype (42 vs. 57 months; log-rank *P*=0.445) (Table 4 and Figure 3A). After adjusting for risk factors in the Cox proportional hazards regression analysis, the MST was comparable between patients with different haplotypes and serum AFP levels. These results indicate that the prognosis of HBV-related HCC patients did not differ significantly between groups. In addition, based on AFP cut-off levels of 20, 200, and 400 ng/ml, lower AFP level was associated with longer MST as compared to higher AFP level (71 vs. 41 months for an AFP cut-off level of 20 ng/ml; 58 vs. 43 months for an AFP cut-off level of 200 ng/ml; and 58 vs. 43 months for an AFP cut-off level of 400 ng/ml) (Table 4), although the difference was not statistically significant.

**Table 4:** Survival analysis of HBV-related HCC patients according to PLCE1 haplotype and serum AFP level

Variables	Patients (n=421)	No. of events (%)	MST (months)	Crude HR(95% CI)	Crude P	Adjusted HR(95% CI)	Adjusted P§
**Haplotypes ψ**							
**AC**	647	283 (46.9)	57	1		1	
**GT**	195	91 (49.5)	42	1.096 (0.865-1.388)	0.449	1.143 (0.900-1.452)	0.272
**AFP (ng/mL)**							
**Cut-off in 20**							
**<20**	119	40 (33.6)	71	1		1	
**≥20**	302	147 (48.7)	41	1.683 (1.186-2.388)	0.004	1.276 (0.887-1.836)	0.19
**Cut-off in 200**							
**<200**	209	86 (41.1)	58	1		1	
**≥200**	212	101 (47.6)	43	1.238 (0.928-1.652)	0.146	0.938 (0.692-1.272)	0.682
**Cut-off in 400**							
**<400**	232	96 (41.4)	58	1		1	
**≥400**	189	91 (48.1)	43	1.262 (0.947-1.683)	0.112	0.985 (0.727-1.335)	0.923

Notes: ψ The number of heplotypes was 2n (2n=842); § Adjustment for Child–Pugh score, tumor size, tumor number, BCLC stage, radical resection, regional invasion, adjuvant antiviral treatment, PVTT in Cox proportional hazards regression model; MST, median survival time; HR, hazard ratio; CI, confidence interval.

### Joint-effects analysis

We further analyzed the association between serum AFP level, *PLCE1* haplotypes, and HBV-related HCC patient survival outcomes. Based on AFP cut-off levels of 200 and 400 ng/ml, lower AFP level with the AC haplotype was associated with a longer MST (Table 5 and Figure 3C and 3D) as compared to other patients. After adjusting for Child–Pugh score, radical resection, antiviral therapy after hepatectomy, tumor size and number, BCLC stage, PVTT, and regional invasion in the Cox proportional hazards regression model, the MST was similar among patients with different AFP levels and haplotypes. For the AFP cut-off level of 20 ng/ml, the MST of *PLCE1* haplotypes combined with AFP level differed significantly. The AC haplotype with AFP < 20 ng/ml had a longer MST (75 vs. 41,71, and 40; log-rank test, *P*=0.0003) (Figure 3B); after adjusting for the above variables, the GT haplotype with AFP < 20 ng/ml was associated with increased risk of death as compared to the AC haplotype (adjusted *P*=0.092, adjusted hazard ratio [HR]=1.539, 95% CI =0.932–2.541) (Table 5). AC and GT haplotypes with AFP ≥ 20 ng/ml had significantly higher risk of death as compared to the AC haplotype with AFP < 20 ng/ml (adjusted HR = 1.392, 95%CI= 1.034–1.873, and adjusted *P* = 0.029 for AC haplotype with AFP ≥ 20 ng/ml; and adjusted HR = 1.445, 95%CI= 1.015–2.057, and adjusted *P* = 0.041 for GT haplotype with AFP ≥ 20 ng/ml) (Table 5).

**Table 5:** Joint-effects survival analysis of PLCE1 haplotypes and serum AFP levels in HBV-related HCC patients

Group	Haplotypes	AFP (ng/mL)	Patients (2n=842)	No. of events (%)	MST (months)	Crude HR(95% CI)	CrudeP	Adjusted HR(95% CI)	Adjusted P§
**I**	**AC**	**AFP <20**	191	59 (30.9)	75	1		1	
**II**	**AC**	**AFP ≥20**	456	224 (49.1)	41	1.826 (1.370-2.433)	0.00004	1.392 (1.034-1.873)	0.029
**III**	**GT**	**AFP <20**	47	21 (44.7)	71	1.417 (0.861-2.332)	0.170	1.539 (0.932-2.541)	0.092
**IV**	**GT**	**AFP ≥20**	148	70 (47.3)	40	1.817 (1.285-2.571)	0.001	1.445 (1.015-2.057)	0.041
**A**	**AC**	**AFP <200**	339	136 (40.1)	61	1		1	
**B**	**AC**	**AFP ≥200**	308	147 (47.7)	51	1.256 (0.994-1.586)	0.056	0.961 (0.751-1.229)	0.749
**C**	**GT**	**AFP <200**	79	36 (45.6)	51	1.124 (0.779-1.624)	0.532	1.282 (0.885-1.858)	0.189
**D**	**GT**	**AFP ≥200**	116	55 (47.4)	40	1.301 (0.951-1.780)	0.100	1.034 (0.750-1.424)	0.839
**1**	**AC**	**AFP <400**	374	150 (40.1)	61	1		1	
**2**	**AC**	**AFP ≥400**	273	133 (48.7)	50	1.286 (1.017-1.624)	0.035	1.007 (0.786-1.290)	0.956
**3**	**GT**	**AFP <400**	90	42 (46.7)	51	1.128 (0.801-1.588)	0.492	1.240 (0.876-1.754)	0.225
**4**	**GT**	**AFP ≥400**	105	49 (46.7)	36	1.318 (0.954-1.821)	0.094	1.077 (0.775-1.499)	0.658

Notes: § Adjustment for Child–Pugh score, tumor size, tumor number, BCLC stage, radical resection, regional invasion, adjuvant antiviral treatment, PVTT in Cox proportional hazards regression model; MST, median survival time; HR, hazard ratio; CI, confidence interval.

### Gene expression omnibus (GEO)data analysis

**Figure 4:**
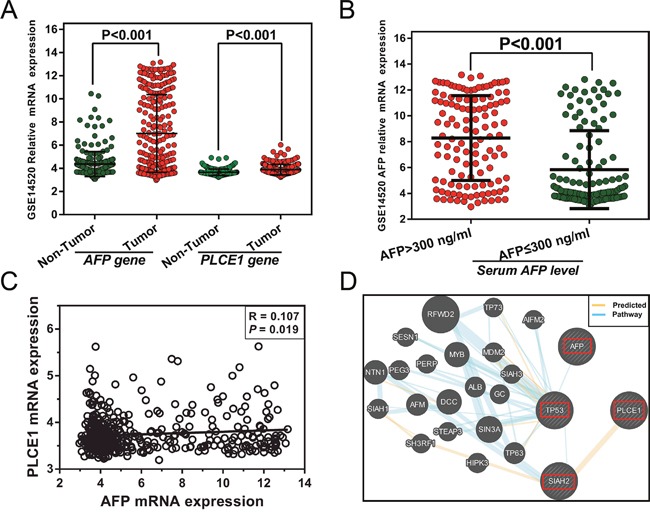
**(A)** *AFP* and *PLCE1* mRNA expression in HBV-related HCC and adjacent normal tissue. **(B)** *AFP* mRNA expression level in HBV-related HCC tumor tissue from different serum AFP level subgroups. **(C)** Correlation between *PLCE1*and *AFP* gene expression levels. **(D)** Gene interaction networks between *PLCE1* and *AFP* genes.

A total of 218 HCC patients from GSE14520 [33] with a history of HBV infection or HBV-related liver cirrhosis were recruited for further analysis. The mRNA expression of *PLCE1*and *AFP* (Affymetrix Probe Set IDs: 205112_at and 204694_at, respectively) differed between HCC and adjacent normal tissues in these patients (*P* < 0.001; Figure 4A), and the latter also differed in tumor tissues of the various serum AFP subgroups (*P* < 0.001; Figure 4B). We also found a weak positive correlation between *PLCE1* and *AFP* mRNA expression in HBV-related HCC tumor tissues (r=0.107, *P*=0.019, Figure 4C). A gene interaction analysis by GeneMANIA predicted that *PLCE1* and *AFP* were involved in the *TP53* pathway (Figure 4D).

**Figure 5:**
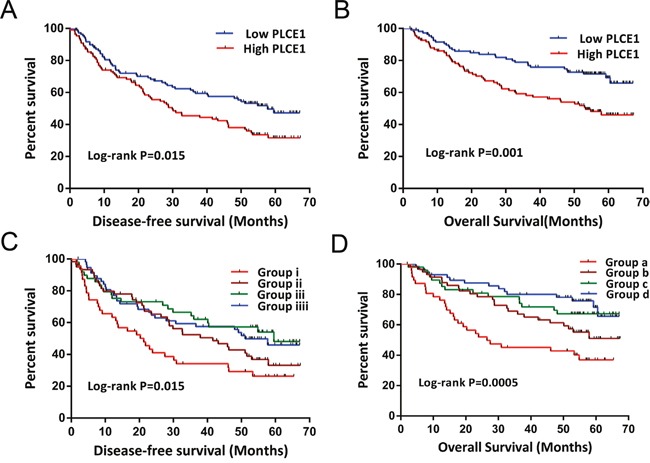
Kaplan-Meier survival curves of GSE14520 HBV-related HCC patient prognosis with different *PLCE1* mRNA expression levels, and joint-effects analysis with different serum AFP levels **(A, B)** DFS **(A)** and OS **(B)** stratified by *PLCE1* expression level. **(C, D)** DFS **(C)** and OS **(D)** stratified by joint-effects analysis of *PLCE1* expression and serum AFP levels.

**Figure 6:**
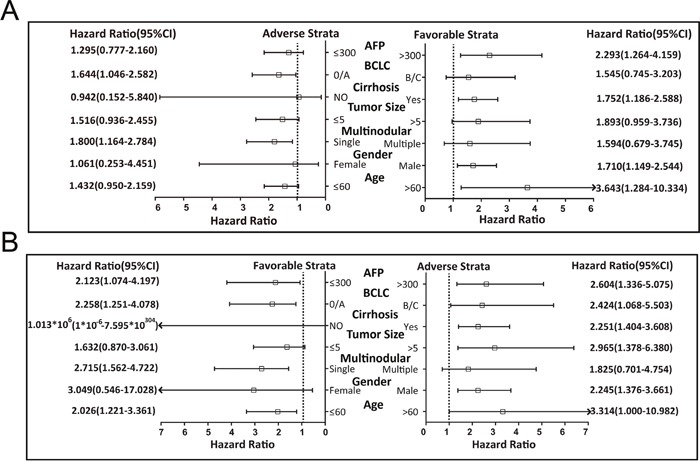
Stratified analysis of the associations between *PLCE1* mRNA expression level and GSE14520 prognosis of HBV-related HCC patients Variables were stratified by favorable and adverse strata. **(A, B)** Stratified analysis between *PLCE1* and DFS **(A)** and between *PLCE1* and OS **(B)**.

The samples were divided into two groups according to *PLCE1* mRNA expression in tumors. The high *PLCE1* group consisted of samples in which *PLCE1* mRNA expression levels were above the median value, with the remaining samples comprising the low *PLCE1* group. Since information on AFP levels were missing for four patients, 214 patients with serum AFP cut-off levels of 300 ng/ml were used for further joint-effects analysis. The results of the survival analysis for *PLCE1* and joint-effect analysis with AFP levels are shown in Tables 6 and 7, respectively. Patients with high *PLCE1* mRNA expression showed poor prognosis (adjusted HR = 1.668, 95%CI= 1.151–2.476, adjusted *P* = 0.007 for disease-free survival [DFS]; adjusted HR = 2.317, 95%CI= 1.448–3.706, adjusted *P* = 0.0005 for OS) (Figure 5A and 5B) as compared to those with low *PLCE1* mRNA expression. Patients with an AFP level > 300 ng/ml had a shorter MST than those with AFP level ≤ 300 ng/ml (35 vs. 48 months, adjusted *P*=0.995), although the difference was not statistically significant. In the stratified analysis, high *PLCE1* expression increased the risk of recurrence and death among patients who were male and had early-stage BCLC, who had a single tumor, cirrhosis, AFP level > 300 ng/ml (Figure 6A and 6B). Old patients with high *PLCE1* expression also had a higher risk of recurrence Figure 6A). Meanwhile, high *PLCE1* expression among young patients with low AFP level and tumor size > 5 cm had an increased risk of death (Figure 6B).

**Table 6:** Survival analysis of PLCE1 mRNA expression and serum AFP levels in HBV-related HCC patients from GSE14520

Variables	Patients (n=218)	DFS				OS			
		No. of events (%)	MST(months)	Adjusted HR(95% CI)	Adjusted P§	No. of events (%)	MST(months)	Adjusted HR(95% CI)	Adjusted P§
**PLCE1 level**									
**Low**	109	52 (47.7)	57	1		31 (28.4)	NA	1	
**High**	109	69 (63.3)	29	1.688 (1.151-2.476)	0.007	53 (48.6)	53	2.317 (1.448-3.706)	0.0005
**AFP (ng/ml)φ**									
**≤300**	118	65 (55.1)	48	1		40 (33.9)	56	1	
**>300**	96	55 (57.3)	35	1.001 (0.693-1.446)	0.995	43 (44.8)	49	1.234 (0.794-1.918)	0.351

Notes: φ Information of AFP was unavailable in 4 patients; § Adjustment for age, gender, cirrhosis, BCLC stage, serum AFP levels; DFS, disease-free survival; OS, overall survival; MST, median survival time; HR, hazard ratio; CI, confidence interval.

**Table 7:** Joint-effects survival analysis of PLCE1 and serum AFP levels in HBV-related HCC patients from GSE14520

Group	PLCE1 level	AFP (ng/ml)	Patients (n=214) φ	No. of events (%)	MST (months)	Adjusted HR(95% CI)	AdjustedP§
**DFS**							
**i**	**High**	**>300**	47	33 (70.2)	21	1	
**ii**	**High**	**≤300**	60	36 (60.0)	41	0.777(0.476-1.266)	0.31
**iii**	**Low**	**>300**	49	22 (44.9)	59	0.459(0.263-0.803)	0.006
**iiii**	**Low**	**≤300**	58	29 (50.0)	51	0.570(0.335-0.968)	0.038
**OS**							
**a**	**High**	**>300**	47	28 (59.6)	26	1	
**b**	**High**	**≤300**	60	25 (41.7)	NA	0.709(0.404-1.246)	0.232
**c**	**Low**	**>300**	49	15 (30.6)	NA	0.384(0.201-0.734)	0.004
**d**	**Low**	**≤300**	58	15 (25.9)	NA	0.347(0.180-0.670)	0.002

Notes: φ Information of AFP was unavailable in 4 patients; § Adjustment for age, gender, cirrhosis, BCLC stage, serum AFP levels; DFS, disease-free survival; OS, overall survival; MST, median survival time; HR, hazard ratio; CI, confidence interval.

The joint-effects analysis of *PLCE1* mRNA expression and serum AFP levels showed that low *PLCE1* expression combined with any serum AFP level was associated with decreased risk of HBV-related HCC recurrence (adjusted HR = 0.459, 95%CI= 0.263–0.803, *P* = 0.006 for patients with low *PLCE1* expression and AFP level > 300 ng/ml; adjusted HR = 0.570, 95%CI= 0.335–0.968, *P* = 0.038 for patients with low *PLCE1* expression and AFP level ≤ 300 ng/ml) (Figure 5C) and death (adjusted HR = 0.384, 95%CI= 0.201–0.734, adjusted *P* = 0.004 for patients with low *PLCE1* expression and AFP level > 300 mg/ml; adjusted HR = 0.347, 95%CI= 0.180–0.670, adjusted *P* = 0.002 for patients with low *PLCE1* expression and AFP level ≤ 300 ng/ml) (Figure 5D) as compared to high *PLCE1* expression and AFP level > 300 ng/ml.

## DISCUSSION

PLCE1 is a member of phosphoinositide-specific PLC family that serves as a link between the second messengers and small GTPases and regulates some Ras family members [34, 35]. The *PLCE1* gene encodes a phospholipase that regulates various processes affecting cell growth, differentiation, and gene expression [36, 37] and was shown to promote intestinal tumorigenesis by inducing inflammation and angiogenesis in a transgenic mouse model [38].

The role of PLCE1 in human cancer remains controversial. It has been suggested to play a tumor suppressor role in and decrease the incidence of colorectal carcinoma (CRC) [39–41]. However, *PLCE1* is thought to act as an oncogene in bladder cancer [42, 43], non-small cell lung cancer [44], skin cancer [45], and head and neck cancer [46]. Upregulation of *PLCE1* mRNA level is associated with longer survival in gastric cardia adenocarcinoma (GCA) and ESCC, while the transcript is downregulated in GCA and ESCC tumor tissue [47], which was confirmed by another study of ESCC patients [30]. However, higher*PLCE1*expression was observed in Chinese Kazakh ESCC patients and ESCC tumor cell lines [48]. In an independent cohort, low PLCE1 expression were linked to poor prognosis in ESCC [47], contradicting a previous report [49]. Our analysis of HBV-related HCC cases from GSE14520 also showed that high *PLCE1* level predicts poor survival and increased risk of recurrence. Gene knockdown studies in ESCC cell lines suggested that *PLCE1* has an oncogenic function in ESCC [49, 50]. In addition, *PLCE1* expression was positively correlated with that of nuclear factor κB-related proteins in Kazakh ESCC patients [51], and negatively correlated with TP53 in HCC and ESCC cells and lung cancer [32, 52, 53]. Distinct microRNAs were shown to suppress *PLCE1* expression and thereby affect cancer development and patient prognosis [44, 49, 54].

Rs2274223 A>G located in exon 26 of the *PLCE1* gene causes a missense mutation (His>Arg) that alters gene expression [29–31]. Multiple case-control studies of Chinese patients revealed that thers2274223is a common susceptibility locus in several cancers, including gastric cancer(GC) [28, 55–58], CRC [59–62], ESCC [28, 30, 31, 37, 58, 63–69], and squamous cell carcinoma of the head and neck [46]. Other studies have reported similar findings for GC and ESCC in other ethnic groups [70–74], and even for gallbladder cancer in an Indian population [75, 76]. However, rs2274223 has not been linked to cancer risk in European CRC patients [77] or northern Indian ESCC patients [78]. Nonetheless, the results of recent meta-analyses indicate that the rs2274223 A >G polymorphism is associated with increased susceptibility to cancer [27, 79]. A survival analysis based on 940 Chinese GC patients revealed that the G allele of rs2274223 was associated with a lower risk of death [80], but this was not true of other cancers. Our results indicate that the G allele of rs2274223 is a cancer risk factor. Rs3765524 C>T also results in a missense mutation in an exon of *PLCE1*, and strong LD with rs2274223 has been reported in several studies [28, 31, 59, 78]. We also detected a strong LD(r^2^=1) between rs2274223 and rs3765524 in the CHB population and Chinese HBV-related HCC patients. A genome-wide association pathway analysis found that rs3765524 contributed to GC susceptibility [81, 82], while a case-control study of the Chinese population showed that rs3765524 was associated with disease risk in GC and ESCC [58, 67, 83]. Investigations of South Asian patients showed similar results for GC [72] but not for ESCC [78, 84]. Additionally, a recent meta-analysis showed that like rs2274223, rs3765524 was significantly associated with disease risk [79]. Given the many factors that can affect tumor incidence and the variable findings in different populations, additional studies are required to evaluate the significance of these polymorphisms in cancer [85].

The *PLCE1* haplotypes examined in the present study were not associated with OS in HBV-related HCC patients. Due to the limited sample size, survival analysis at three AFP cut-off levels showed no differences after adjustment in a COX proportional hazards regression model, contrary to the findings of a recent study [22]. Our results suggest that both the genotypes and haplotypes of rs2274223 and rs3765524 were significantly associated with HBV-related HCC incidence at AFP cut-off levels of 200 and 400 ng/ml. The GT haplotype had an increased risk of high AFP level in HBV-related HCC as compared to the AC haplotype. We also observed similar clinical outcomes in HCC patients grouped by haplotype and AFP cut-off levels of 200 and 400 ng/ml. In addition, according to an AFP cut-off level of 20 ng/ml, the distribution of *PLCE1* genotypes and haplotypes did not reach statistical significance in HBV-related HCC. However, a significant interaction between *PLCE1* haplotypes and a serum AFP cut-off level of 20 ng/ml was observed in a joint analysis. Grouping by *PLCE1* haplotype and an AFP cut-off level of 20 ng/ml, the outcomes of patients with AC or GT and AFP level ≥ 20 ng/ml differed from those with AC haplotypes and AFP level< 20 ng/ml. These findings demonstrate that *PLCE1* gene polymorphisms combined with serum AFP level can serve as a prognostic marker for HBV-related HCC patients treated by hepatic resection. Once validated, *PLCE1* haplotypes of rs2274223 and rs3765524 may be used in combination with other clinical prognostic factors for decision-making in HCC management.

GEO data analysis of HBV-related HCC suggested that *PLCE1*mRNA expression was upregulated in tumor tissue, which predicted poor prognosis. These results imply that *PLCE1* acts as an oncogene in HBV-related HCC, and may be a potential therapeutic target. The stratified analysis also revealed that high *PLCE1* expression increased the risk of recurrence and death in patients who were male and had early-stage BCLC, a single tumor, cirrhosis, and AFP level > 300 ng/ml. In addition, older patients had an increased risk of recurrence, whereas younger patients with low AFP level and tumor size > 5 cm had a higher risk of death. These findings suggest that higher *PLCE1* expression was associated with tumor progression and degree of malignancy, which in turn affects clinical outcome. A recent report indicated that serum AFP is a valuable prognostic biomarker in HBV-related HCC and that lower preoperative serum AFP levels were associated with a much higher OS rate [22]. Our previous study of HBV-related HCC also demonstrated that serum AFP level was associated with 2-year OS and RFS, but was not useful for predicting long-term survival and recurrence in HBV-related HCC [86], which requires a combination of serum AFP and other markers. Indeed, in the present study the combination of serum AFP and *PLCE1* expression showed a strong interaction and better predictive value for HBV-related HCC prognosis.

In conclusion, *PLCE1* gene polymorphisms are associated with high AFP level(≥ 200 or 400ng/ml) in HBV-related HCC and can predict OS of patients following hepatic resection by stratification according to a serum AFP level of 20 ng/ml. In addition, we found that *PLCE1* mRNA expression combined with serum AFP stratified at 300 ng/ml can predict HBV-related HCC prognosis and recurrence. Thus, the *PLCE1* gene is a potential prognostic marker in HBV-related HCC, especially combined with serum AFP level. This is the first study reporting that the combination of serum AFP and *PLCE1* gene polymorphism and mRNA expression has significant predictive value for clinical outcome of HBV-related HCC patients. Our findings provide evidence for the value of *PLCE1*gene polymorphism and mRNA expression in distinguishing the morbidity and prognosis of different subgroups of HBV-related HCC, and provide a basis for the development of personalized treatment strategies.

## MATERIALS AND METHODS

### Study population

The study protocol was approved by the Ethics Committee of the First Affiliated Hospital of Guangxi Medical University (approval no. 2015KY-E-032). Fresh surgically resected and pathologically confirmed HCC specimens (n = 421) collected at the First Affiliated Hospital of Guangxi Medical University from 2001 to 2013 were analyzed. All patients were positive for serum HBV surface antigen. Serum AFP levels were measured before hepatectomy. Cancer tissue specimens were collected during surgery and immediately stored at −80°C until use.

### Genotyping

Genomic DNA was extracted from tumor samples using the TIANamp Genomic DNA kit (Tiangen Biotech, Beijing, China). Samples were genotyped by DNA sequencing with an ABIPrism 3100 system (Applied Biosystems/Shanghai Sangon Biological Engineering Technology and Services, Shanghai, China) using the following forward and reverse primers: 5′-GTTCTTGGGATTCCTTTGC-3′ and 5′-CA TGGGTGAGGCTGTACTTT-3′ for rs2274223; and 5′-GCTATGACTGTTTACTGGGATG-3′ and 5′-AAG GAGCGAGGTGAGCAT-3′ for rs3765524. Sequencing results were analyzed using Chromas software (http://technelysium.com.au/wp/chromas/) under conditions where signal-to-noise ratio was > 98%.

### Association analysis

Hardy-Weinberg equilibrium was estimated for each SNP with the goodness-of-fit χ^2^ test. Linkage disequilibrium (LD) between SNPs of the *PLCE1* gene was calculated using Haploview v.4.2, and the LD of normal CHB population was calculated based on published HapMap genotype data (https://snpinfo.niehs.nih.gov/snpinfo/snptag.html). Binary logistic regression was used to analyze the genetic model of *PLCE1* genotypes and haplotypes for different serum AFP levels and the association between clinical risk factors and *PLCE1* haplotypes. To investigate the association between *PLCE1* mRNA expression and serum AFP level in the prediction of HBV-related HCC patient survival, we analyzed the expression profile chip dataset of HBV-related HCC from GEO (GSE14520). GeneMANIA was used for gene interaction analysis.

### Statistical analysis

The correlation between AFP level and *PLCE1* gene polymorphisms was assessed with the Spearman correlation coefficient. ORs and corresponding 95% CIs were calculated to estimate relative risk in the binary logistic regression model. Survival analysis was performed using the Kaplan-Meier method with the log-rank test for different clinical factors and haplotypes. Cox proportional hazards regression analysis was used to calculate the crude or adjusted HRs and 95% CIs in uni-and multivariate analyses, with adjustment for those with P < 0.1 in the univariate analysis or selected variables. A *P* value < 0.05 was considered statistically significant. Statistical analyses were carried out using SPSS v.20.0 software (IBM, Chicago, IL, USA).

## SUPPLEMENTARY MATERIALS FIGURES



## Abbreviations

PLCE1: phospholipase C epsilon 1
AFP: α-fetoprotein
HBV: hepatitis B virus
HCC: hepatocellular carcinoma
SNPs: single nucleotide polymorphisms
CHB: Chinese Han in Beijing
OS: overall survival
DFS: disease-free survival
AFB1: aflatoxin B1
TP53: tumor protein p53
BCLC: Barcelona Clinic Liver Cancer
PVTT: portal vein tumor thrombus
LD: linkage disequilibrium
MST: median survival time
OR: odds ratio
HR: hazard ratio
CI: confidence interval
