# Supplementary material for: PLCE1 polymorphisms and expression combined with serum AFP level predicts survival of HBV-related hepatocellular carcinoma patients after hepatectomy
Source: Oncotarget. 2017 Mar 18;8(17):29202–19. doi: 10.18632/oncotarget.16346 (PMC5438724; doi:10.18632/oncotarget.16346)
Supplement: Supplementary file 1 [file oncotarget-08-29202-s001.pdf]

PLCE1 polymorphisms and expression combined with serum AFP level predicts survival of HBV-related hepatocellular carcinoma patients after hepatectomy

SUPPLEMENTARY MATERIALS

SUPPLEMENTARY FIGURES

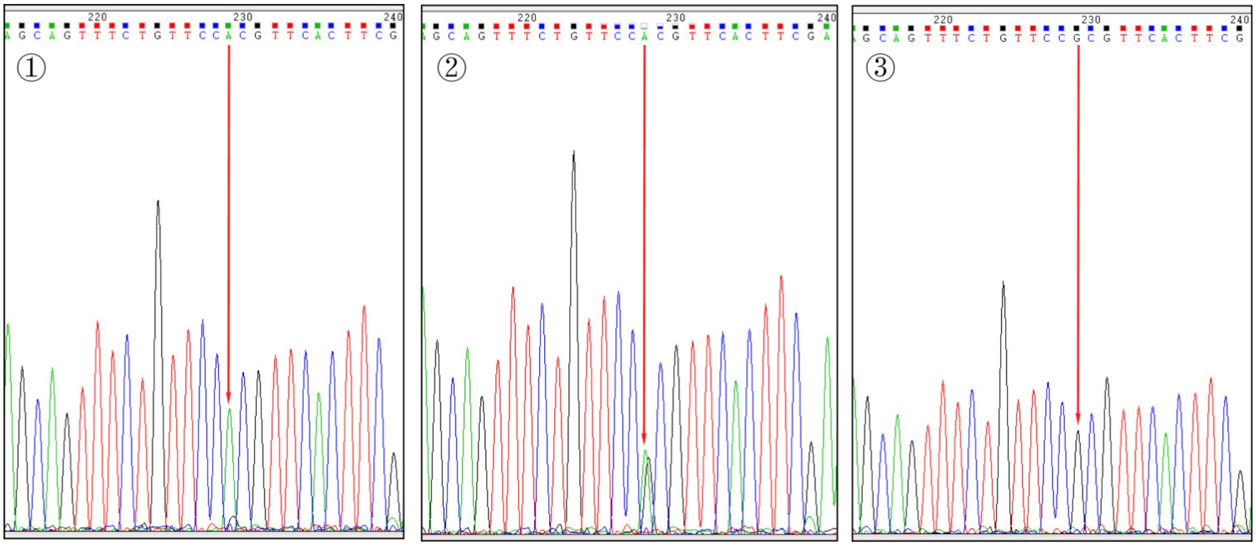

Supplementary Figure 1: Sequencing map for genotypes of PLCE1 rs2274223 polymorphism. The arrows in ①–③ show AA, AG, and GG genotypes, respectively

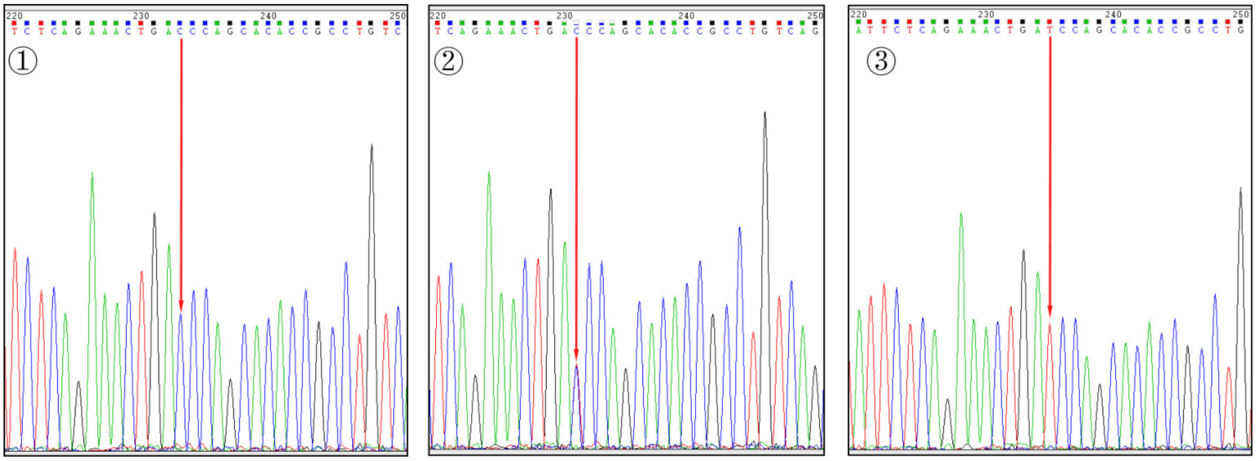

Supplementary Figure 2: Sequencing map for genotypes of PLCE1 rs3765524 polymorphism. The arrows in ①–③ show CC, CT, and TT genotypes, respectively.
